# Behaviour of Endothelial Cells in a Tridimensional *In Vitro* Environment

**DOI:** 10.1155/2015/630461

**Published:** 2015-02-19

**Authors:** Raif Eren Ayata, Stéphane Chabaud, Michèle Auger, Roxane Pouliot

**Affiliations:** ^1^Centre de Recherche en Organogénèse Expérimentale de l'Université Laval/ LOEX, Québec, QC, Canada G1J 1Z4; ^2^Division of Regenerative Medicine, CHU de Québec Research Centre, Quebec, QC, Canada G1J 1Z4; ^3^Faculté de Pharmacie, Université Laval, Québec, QC, Canada G1V 0A6; ^4^Département de Chimie, PROTEO, CERMA, Université Laval, Québec, QC, Canada G1V 0A6

## Abstract

Angiogenesis is a fundamental process in healing, tumor growth, and a variety of medical conditions. For this reason, *in vitro* angiogenesis is an area of interest for researchers. Additionally, *in vitro* angiogenesis is important for the survival of prevascularized tissue-engineering models. The aim of this study was to observe the self-tubular organization behaviour of endothelial cells in the self-assembly method. In this study, bilayered and dermal substitutes were prepared using the self-assembly method. Histological, immunostaining, and biochemical tests were performed. The behavioural dynamics of endothelial cells in this biological environment of supportive cells were observed, as were the steps of the *in vitro* angiogenic cascade with self-organizing capillary-like structures formation. The epidermal component of the substitutes was seen to promote network expansion and density. It also increased the quantity of angiogenic factors (VEGF and Ang-1) without increasing the proinflammatory factor (IL-8). In addition, the increased MMP activity contributed to matrix degradation, which facilitated capillary formation.

## 1. Introduction

Endothelial cells (ECs) lining the vascular tree form a strict monolayer of flattened noncycling (quiescent) cells [1]. Angiogenesis is a complex biological process involving the activation of ECs and the outgrowth of new blood vessels from existing vessels. The activated cell (tip cell) migrates towards stimuli in the extracellular matrix by degrading the matrix. Adjacent cells (stack cells) begin to proliferate and follow the leader cell. Afterwards, a capillary sprout and lumen formation take place, and these then mature into a newly formed capillary [2, 3].

Angiogenesis takes place during normal physiological processes such as ovulation, embryonic development, and wound healing [4]. However, angiogenesis is also seen in pathological conditions such as cancer, psoriasis, diabetes, and arthritis [5]. Its presence in both healthy and pathological situations makes angiogenesis an intriguing area of research [6]. The formation of capillary-like tubes (CLTs) by ECs has been undertaken in* in vitro* assays [7, 8] as well as in tissue-engineering applications [9]. In the context of tissue engineering, the main objective is the survival of grafts after transplantation [9]. In* in vitro* assays, the investigation focuses on the mechanistic aspects of capillary morphogenesis in a controlled environment [10]. There are numerous types of assays, but most involve EC suspensions with or without supportive cells [6] in either a three-dimensional extracellular matrix gel such as fibrin [11–13] or collagen [11, 14, 15], or in polymer-based scaffolds [16–19] and three-dimensional engineered tissues [9, 20, 21].

The methods mentioned above have shown that formation of CLTs* in vitro* generally relies on the self-organization of ECs, either by themselves or with supportive cells. Media supplements have been seen to contribute to the proliferation and maintenance of cell morphology and phenotype. Rochon et al. (2010) [21] studied the effect of epithelial cells on the size of CLTs. They observed that keratinocytes were able to regulate CLT size and morphology. Rochon's bilayered skin model showed smaller and regular CLTs when compared with dermal models without keratinocytes. In addition, when they seeded keratinocytes onto the dermal models, they observed reversible sizes and morphological changes. Another research group, Liu et al. (2013) [22], investigated the effect of keratinocytes on the vascularization pattern of* in vitro* skin models. They determined that there were more capillaries in bilayered skin models than in dermal models. Also, the expression of angiogenic factors was increased in bilayered skin models compared to each component of the model. They therefore concluded that keratinocytes played a direct or indirect role in the vascularization process of* in vitro* models.

The goal of our study was to construct CLTs using a multicellular culturing technique to observe the consecutive steps of angiogenesis in a bilayered skin substitute reconstructed by the self-assembly method and to see how keratinocytes affect the behaviour of CLTs during angiogenesis in this model.

## 2. Materials and Methods

### 2.1. Cell Culture

Fibroblasts, keratinocytes, and human dermal microvascular endothelial cells (HDMECs) were isolated from samples of healthy breast skin. The keratinocytes (≈5000 cells/cm^2^) were seeded onto a feeder layer of irradiated 3T3 mouse fibroblasts (20000 cells/cm^2^); passage 4 fibroblasts and passage 1 keratinocytes were used in all experiments. The HDMECs were isolated from the dermis by scrubbing and applying pressure to the dermal pieces. The HDMECs were then purified using anti-human CD31 dynabeads (Invitrogen). The concentration of magnetic dynabeads varied between 3 : 1 and 1 : 3 bead-cell ratio depending on the density of fibroblast-cell contamination in the sample. The HDMECs were then cultured on gelatin coated 6-well plates. HDMECs from passage 2 were used for all experiments.

DME (Dulbecco-Vogt modification of Eagle's) medium with supplements (10% fetal calf serum, penicillin (P, 100 IU/mL), and gentamicin (G, 25 *μ*g/mL)) was used for the fibroblasts. Keratinocytes were cultured in DH (Dulbecco-Vogt modification of Eagle's medium with Ham's F12 in a 3 : 1 ratio) with supplements (5% FetalClone II serum, 5 *μ*g/mL insulin, 0.4 *μ*g/mL hydrocortisone, 10 ng/mL epidermal growth factor, 10^−10^ mol/L cholera toxin, P, and G). EGM-2 MV SingleQuots Kit media with supplements and growth factors (Lonza) were used for ECs. Culture media were changed three times a week. All cultures were kept under 8% CO_2_ and at 37°C in an incubator.

### 2.2. Assessment of Endothelial Cell Culture Purity

The cultured HDMECs were harvested using trypsin and subjected to immunofluorescence staining with CD31- (Invitrogen, 1/100) Alexa 488 antibody. They were then passed through flow cytometry to ascertain purity.

### 2.3. Preparation of Endothelialized Bilayered Skin and Dermal Substitutes with Self-Assembly Method

**Figure 1:**
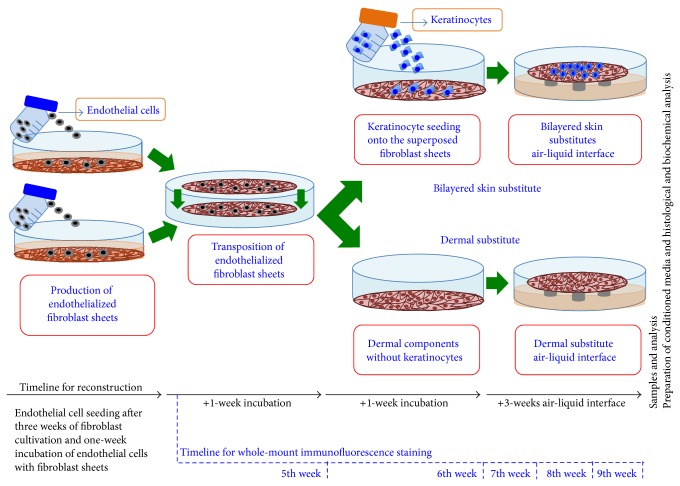
Self-assembly method and experimental timelines. The skin substitutes were produced using the self-assembly method. The fibroblasts were cultured in the presence of ascorbic acid, and then ECs were seeded onto the fibroblast sheets. The manipulable sheets were transposed and incubated for 7 days to form the dermal component. Keratinocytes were seeded upon endothelialized fibroblast sheets to prepare bilayered skin substitutes. After one week of culture, the dermal and bilayered substitutes were raised to the air-liquid interface.

Fibroblast cells (8000 cells/cm^2^) were seeded onto the plates and cultured for three weeks with DMEM freshly supplemented with ascorbic acid (50 *μ*g/mL). At the end of this period, ECs (12,000 cells/cm^2^) were seeded onto those fibroblast sheets and cultured for an additional week in a mixed medium (1 : 1, DMEM: EGM-2 MV SingleQuots Kit media with supplements, growth factors, and ascorbic acid). Pairs of endothelialized fibroblast sheets were then clipped together to produce the dermal part of the substitutes and cultured with mixed media for one week to fuse the two sheets. At the end of the fourth week, keratinocytes (300,000 cells/cm^2^) were seeded onto the fused fibroblast sheets and cultured for one week under the same culture conditions except the fact that the medium was a 1 : 1 mixture of DH and EGM-2 MV SingleQuots Kit media with supplements, growth factors, and ascorbic acid. The dermal substitutes were treated in the same way as the bilayered skin substitutes to ensure all substitutes experienced the same conditions. Afterwards, the keratinocyte seeded and the unseeded dermal substitutes were brought to the air-liquid interface and cultured in the mixed medium (1 : 1, DH without the EGF: EGM-2 MV SingleQuots Kit media with supplements, growth factors, and ascorbic acid) for three weeks (Figure 1).

### 2.4. Histology

*Masson's Trichrome Staining*. Biopsies from different areas of each substitute were fixed overnight in Histochoice solution and embedded in paraffin wax. 5 *μ*m thick sections were cut and stained with Masson's trichrome.

*Immunohistochemistry (IHC)*. This was achieved with 5 *μ*m thick OCT sections. Anti-human VEGF antibody (Santa Cruz, 1 : 100) and IHC reagent kits from Vector Laboratories were used. Nucleus staining was not performed.

*Immunofluorescence (IF)*. Staining was done on confluent monolayer HDMECs. Cells were fixed with 90% acetone (10 min) and then were treated with human CD31 (1/100, Invitrogen, 45 min) and Alexa 488 + Hoechst 33258 (30 min) and covered with mounting medium on slides.

### 2.5. Preparation of Conditioned Media

The conditioned media (CMs) were collected from monolayer fibroblasts and keratinocytes cultures, as well as from the bilayered and dermal substitutes. The conditioned media were harvested from monolayer cells when they reached 80–90% confluency in 75 cm^2^ cell culture flasks and from substitutes at the end of the air-liquid interface phase. The medium was pipetted and discarded, and the flasks and substitutes were washed gently three times with serum-free and additive-free medium. The cells and substitutes were then cultured for another 48 hours with 10 mL of medium. The CM fractions were centrifuged and stored at −80°C prior to the endothelial cell proliferation and organization assay, the enzyme linked-immune sorbent assay (ELISA), and the matrix metalloproteinase (MMP) assay.

### 2.6. Endothelial Cell Proliferation and Organization Assay

Equal numbers of HDMECs were seeded onto 6-well plates and cultured with EGM-2 MV SingleQuots Kit media with supplements and growth factors for 24 hours. The cells were then washed gently with the appropriate medium (keratinocyte CM, fibroblast CM, or a mix of both or EGM-2 MV SingleQuots Kit media with supplements and growth factors) and incubated with the same medium for four additional days. The medium was changed every 48 hours. At the end of four days, cells were observed with a light microscope. They were then trypsinized, centrifuged, and counted.

### 2.7. ELISA, Western Blot, and MMP Analyses with Bilayered and Dermal Substitutes

VEGF, IL-8, and Ang-1 expressions in CM were measured with ELISA kits from R&D systems. Total MMP activity in CM was assessed with the Sensolyte fluorimetric MMP assay kit from AnaSpec. Western blot analysis was performed with 30 mg total protein of substitute lysates per well with acrylamide gel for SDS-PAGE, and the proteins were transferred to a PVDF (Biorad) or cellulose (Amersham) membrane using an electrical current. The membrane was incubated with primary antibody anti-human collagen-I (Rockland) and an appropriate peroxidase conjugated secondary antibody (Thermo Scientific). The protein levels were determined using SuperSignal West Pico Chemiluminescent Substrate (Thermo Scientific).

### 2.8. Whole-Mount Immunofluorescence Staining

Endothelial cell seeding takes place after 3 weeks of fibroblast-only culture. The behaviour of endothelial cells was then observed on a weekly basis, using whole-mount immunofluorescence staining for five weeks (ending in the ninth week) (Figure 1). The procedure of endothelialization took five weeks in total. Therefore, whole-mount immunofluorescence staining was completed each week, using fresh substitutes. Each week, the substitutes were fixed in ice-cold acetone (4°C) and then were treated with a primary antibody (CD31, Invitrogen; 1 : 100) and a secondary antibody mixture (Alexa 488, Invitrogen; 1 : 400 + Hoechst 33258 (Sigma-Aldrich; 1/100)). Each step took 24 hours, with the substitutes being washed several times with PBS between each step. Finally, the substitutes were slided and covered with mounting medium and coverslips. The slides were nail-polished and observed with a Zeiss LSM 700 laser-scanning confocal microscopy system.

### 2.9. Statistics

The significance of the data was determined by the Mann-Whitney *U* or Kruskal-Wallis test using SPSS 13 software. A value of *P* < 0.05 was accepted as significant.

## 3. Results

### 3.1. Endothelial Cell Culture Purity

**Figure 2:**
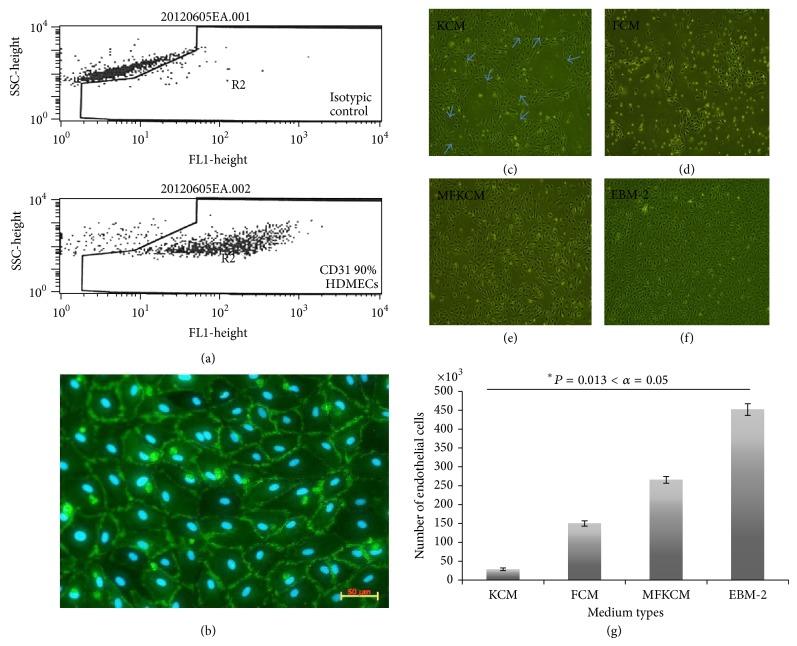
Endothelial cell purity and effect of conditioned medium from monolayer cultures. (a) Flow cytometry results of HDMECs. (b) CD31-Alexa 488 (green) IF staining of monolayer HDMECs (20x magnification, scale bar = 100 *μ*m). Effect of conditioned media (CM) on endothelial cell monolayer culture organization and physiology on four-day-old culture. (c) Monolayer keratinocyte conditioned medium (KCM); circular branch-like structures were indicated with blue arrows. (d) Monolayer fibroblast conditioned medium (FCM). (e) Mixture of fibroblast and keratinocyte conditioned medium (MFKCM, 1 : 1). (f) EGM-2 MV SingleQuots Kit media with supplements and growth factors (EBM-2). (g) HDMECs proliferation assay with CM.

The first step in working on* in vitro* angiogenesis was to be sure that the endothelial cell culture was as pure as possible. The endothelial cells were purified with CD31 labeled magnetic dynabeads. 90% CD31 positive ECs were observed by flow cytometry (Figure 2(a)) compared to isotypic control and the classic cobblestone morphology of ECs was observed with CD31 immunofluorescence staining (Figure 2(b)). As previously noted, the purification of ECs was improved by adjusting the bead : cell ratio. In case of high fibroblast contamination, decreasing the bead-cell ratio to 1 : 3 eliminated the contamination more effectively.

### 3.2. Keratinocytes Conditioned Medium Induces Reorganization of a Monolayer Endothelial Cell Culture

In order to determine if keratinocytes could enhance the formation of CLT, tests on monolayer cultures were conducted using CM from keratinocyte or fibroblast cultures or a mix of both. Commercial EGM-2MV was used as control. The effect of CM on endothelial cell physiology on four-day-old culture showed that the keratinocyte conditioned medium (KCM) induced HDMECs to organize and differentiate cells to construct circular patterns. It is probable that the KCM also directed the ECs to develop branches (Figure 2(c), blue arrows). When this happened, EC proliferation ceased and there was a net decline in the number of ECs found when they were counted (Figure 2(g)). However, it was noted that the fibroblast conditioned medium (FCM) induced cell proliferation, although dead cells were also observed (Figures 2(d) and 2(g)). And, finally, the mixture of fibroblast and keratinocyte conditioned medium (1 : 1, MFKCM) was seen to induce the HDMECs to proliferate (Figures 2(e) and 2(g)), but few circular structures were observed which means branch formation was delayed. As seen in the diagram, EGM-2 MV SingleQuots Kit media (EBM-2) was the most effective medium for inducing the proliferation of HDMECs (Figure 2(g)). However, no circular organizations were seen in monolayer HDMEC cultures when EBM-2 was used (Figure 2(f)). Therefore, EBM-2 alone did not induce HDMECs to form branches even when angiogenic growth factors and supplements were present (Figure 2(f)).

### 3.3. Histological Architecture

**Figure 3:**
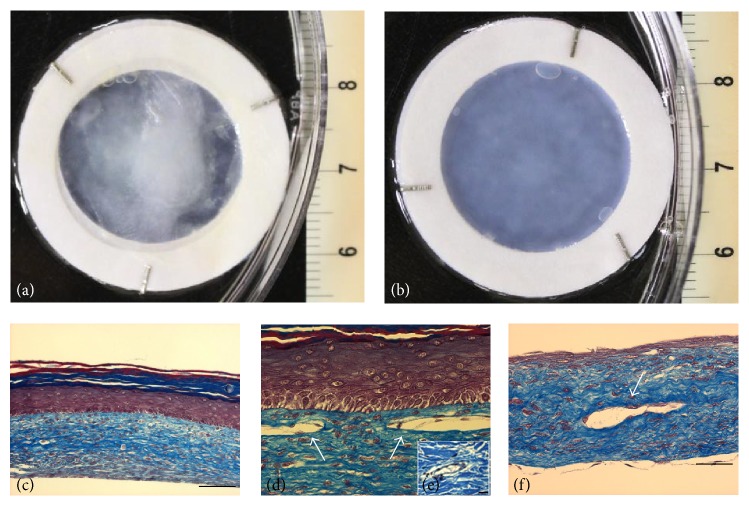
Histological analysis. Macroscopic appearance. (a) Bilayered skin model (epidermis + dermis). (b) Dermal model (without epidermis), histological analysis, and Masson's trichrome staining. (c) Bilayered skin model (10x magnification, scale bar = 100 *μ*m). (d) Endothelialized bilayered skin model (20x magnification). (e)* Ex vivo* skin capillary (40x magnification, scale bar = 20 *μ*m). (f) Dermal model (20x magnification, scale bar = 50 *μ*m). Capillary-like tubes (CLTs) are indicated with arrows.

After the three-week air-liquid interface phase, macroscopic analysis of skin substitutes showed that the bilayered substitutes had a whitish surface due to the presence of the multilayered corneocytes (Figures 3(a) and 3(b)). Histological findings of these substitutes showed the formation of a dense dermis. The dermis was made up of a collagen matrix containing numerous fibroblast cells (Figures 3(c), 3(d), and 3(f)). A well-stratified epidermis and several cornified layers were observed on the top of the keratinocyte-seeded substitutes (Figures 3(c) and 3(d)). The transition in cell morphology from a cuboidal and/or columnar shape in the basal layer to a flattened shape in the corneum layer was clearly visible (Figure 3(d)). Additionally, keratinocytes appeared to have lost their nucleus in the uppermost layers (Figure 3(d)). As expected, HDMEC-seeded substitutes had evidence of self-reconstruction of the CLTs showing as ring-like structures (Figures 3(d) and 3(f)), similar to the one present in native skin (Figure 3(e)), and they were beset by ECs (Figures 3(d) and 3(f)).

### 3.4. Self-Organization of Capillary-Like Structures

**Figure 4:**
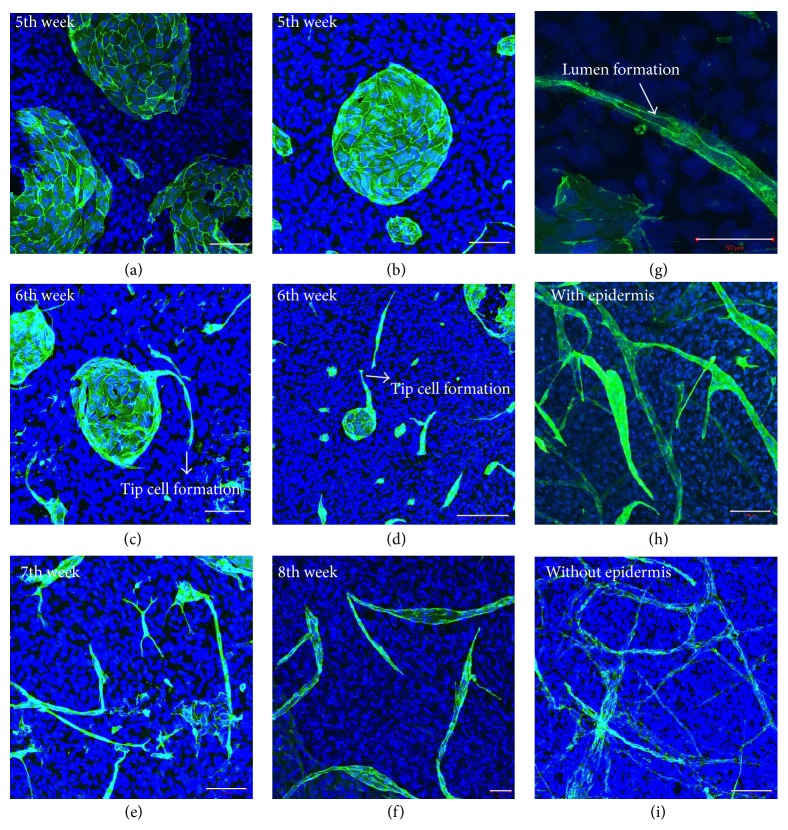
Observation of endothelial cell behaviour with whole-mount immunostaining. The steps of capillary formation in the* in vitro* skin model on a weekly basis after EC seeding: (a, b) fifth week, (c, d) sixth week, (e) seventh week, (f) eighth week, (g) lumen formation (40x magnification), and (h, i) ninth week, the end of the procedure (all figures (except (g)) were captured at 20x magnification with confocal microscopy) ((scale bar = 100 *μ*m for (a), (b), (c), (e), (h), and (i)) – (scale bar = 50 *μ*m for (f) and (g)) and scale bar = 200 *μ*m for (d)).

At the end of three weeks, in the culture of dermal fibroblasts with ascorbic acid supplementation, the fibroblasts had secreted their own biological matrix. The cells also released factors into this matrix, which resulted in HDMECs having endothelial cell-matrix interactions and fibroblast-endothelial cell contact. However, the endothelialized fibroblast sheets were not sufficiently manipulable at this stage, so the sheets were transposed one week later. At that time, a whole-amount IF staining procedure was undertaken and redone on a weekly basis thereafter. Samples from the fifth week showed that HDMECs had constructed large and small colonies on the fibroblast sheets (Figures 4(a) and 4(b)). The following week (sixth week), we observed that HDMEC colonies had started to breakdown, and a leader cell formation (tip cell) from the colonies was seen to have penetrated the fibroblast-secreted matrix (Figures 4(c) and 4(d)). In addition, we observed that the cells around the colonies had self-organized, lengthened, and stretched to follow the leader cell. In the ensuing weeks (seventh and eighth weeks), the breakdown of the colonies continued and the formation of long tubular structures was observed (Figures 4(e) and 4(f)). Lumen formation was also seen in a CD31 labelled tubular structure (Figure 4(g)). The presence of lumens was confirmed by serial histological cross sections. At the end of the procedure (ninth week), a complex capillary network formation was evident. The eagle-eye observation of substitutes showed tubular density differences between the samples with epidermis and samples without (Figures 4(h) and 4(i)). The bilayered substitutes (Figure 4(h)) displayed more branched, complex, and numerous CLTs than their dermal counterparts (Figure 4(i)).

### 3.5. Epidermis Favours Construction of Capillary-Like Structures

**Figure 5:**
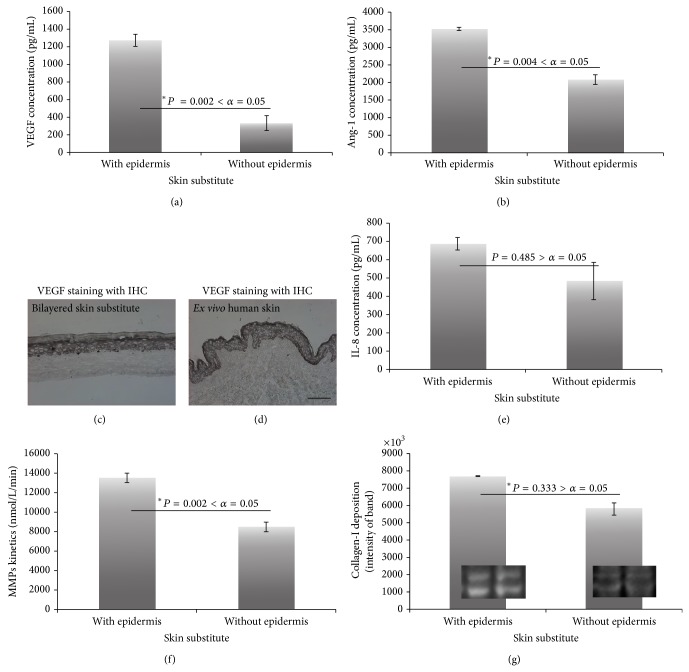
Biochemical analysis with the conditioned media of substitutes. ELISA (*n* = 5 or 6), MMP activity (*n* = 6), and western blot results. ELISA results of (a) VEGF and (b) Ang-1. VEGF staining with IHC of (c) bilayered skin model and (d)* ex vivo* human skin (10x magnification, scale bar = 100 *μ*m). (e) ELISA results of IL-8. (f) MMP activity analysis of bilayered and dermal substitutes and (g) collagen-I deposition of substitutes.

The increased* in vitro* angiogenesis by epidermis was shown with three different angiogenic stimuli. The CM (without any supplements, growth factors, or serum) of bilayered substitutes showed increased expression of two kinds of angiogenic growth factors (vascular endothelial growth factor (VEGF) and angiopoietin-1 (Ang-1)) (Figures 5(a) and 5(b)). In addition, the dominant expression of VEGF by epidermis was confirmed by IHC staining, where it was found that the staining pattern was intense in the epidermal component of substitutes (Figure 5(c)) as well as of* ex vivo* skin (Figure 5(d)). These results were consistent with ELISA results (Figure 5(a)). However, the presence of epidermis did not affect the proinflammatory cytokine (interleukin-8 (IL-8)) release (Figure 5(e)). This means the increased CLT formation in bilayered models is not due to inflammation.

MMP activity and matrix degradation are important factors in angiogenesis. The total MMP activity, as determined by fluorescence, followed a similar pattern to the angiogenic factors. Hence, there was increased MMP activity in bilayered models (Figure 5(f)). The next step in the evaluation was the western blot analysis to determine whether collagen was present in the substitutes. Attempts were made to quantify deposited collagen in the matrix using Bio 1D optical density analysis software with western blot results. No significant difference was found between bilayered and dermal substitutes despite increased MMP activity in bilayered substitutes. It was speculated that there was more matrix modeling in bilayered substitutes to help ECs with branch formation (Figure 5(g)).

## 4. Discussion 

Angiogenesis is a key mechanism in a variety of normal physiological processes as well as an array of pathological conditions. It is controlled by proangiogenic and antiangiogenic factors and, under normal circumstances, these factors are in balance. In pathological conditions, however, this is not the case, and increased or impaired angiogenesis can occur. To study such things as the mechanisms, genetics, and signal pathways of angiogenesis,* in vitro* and* in vivo* angiogenesis assays have been developed. The purpose of this paper is to report on our observations of EC behaviour with regard to capillary-like network expansion, in a spatial and well-controlled* in vitro* biological environment. Our investigation of the medium and epithelial-cell effect on angiogenesis was undertaken. The skin model served as the environment, and HDMECs were chosen since different subsets of ECs have different responses and behaviours.

The technical challenges of the vascularization in tissue-engineered organ models were well explained by Auger et al. [23]. They stated that EC proliferation, sprout interconnection, cell-cell contact, and stimulating factors are all necessary for constructing mature and stable capillaries. In addition, they indicated that the survival of endothelial cells is essential for the maintenance of vascular integrity. For this reason, we undertook a study of CM and found that maximum EC proliferation could be obtained with EGM-2 MV SingleQuots Kit media (EBM-2) with supplements and growth factors. Therefore, it was decided to mix this medium with other media because ECs were planned to be seeded. Previously, Gibot et al. [20] and Rochon et al. [21] completed similar studies using a 1 : 1 mixed-medium system for just two weeks. But when the mixed medium was used for two weeks, no network formation or expansion was obtained in the substitutes in this study (data not shown). With present methodology, the ECs were induced to proliferate with angiogenic supplements and growth factors in EGM-2 MV SingleQuots Kit media (EBM-2) and to construct complex vascular networks with the help of fibroblasts and fibroblast-secreted extracellular matrix. This matrix immobilized angiogenic factors, created an excellent environment for ECs, and allowed epithelial cells (keratinocytes) to function as a scaffold. Moreover, the absence of CLTs in the two-week mixed-medium method demonstrated that the secreted factors of fibroblasts and keratinocytes are insufficient for the formation of complex CLTs applicable to our self-assembly method and model system. Therefore, mixing EGM-2 MV SingleQuots Kit media with other media is necessary.

The primary function of vessels is to provide blood circulation. Therefore, lumen formation is essential for sprouting angiogenesis. There are two mechanisms for lumen formation: cell hollowing and cord hollowing [24]. The lumen formation could be observed using histological and IF staining but not the mechanism of cell or cord hollowing, an unfortunate short-coming of our model. Montaño et al. [13] explained the intracellular vacuole formation mechanism by pinocytosis and the fusion of vacuoles over time with their fibrin gel model, because fibrin gel models are transparent structures to observe with light microscopy. They also found that long, continuous lumen formation was rarely seen* in vitro*. It should be noted that a disadvantage of our method compared to the gel model is the absence of light microscopic observations, because the individual angiogenic steps could not be observed with an ordinary light microscope for transparency reasons. IF studies had to be used for each phase, which had cost implications. Alternatively, virus transfected fluorescent ECs would be used, as Gibot et al. [20] did, to observe the steps, but virus transfection also brings other costs and risks such as changes in EC phenotype.

In contrast to Rochon et al. [21], we obtained larger CLTs in bilayered substitutes than in the dermal substitutes. In addition, the CLTs in our bilayered substitutes were more complex, dense, and branched than in our dermal substitutes. Liu et al. [22] had the same results. It was the increased VEGF and Ang-1 expression (the master regulators of angiogenesis) that resulted from seeding with keratinocytes that enhanced CLT formation. This is because these factors play an important role in* in vitro* endothelial cell survival, proliferation, and network expansion [25, 26]. IL-8 was used as a marker of inflammation and irritation to evaluate topical products on skin substitutes [27]. Analysis for its secretion in the substitutes was completed. Because keratinocyte seeding did not contribute to increase expression of proinflammatory cytokine IL-8, inflammation was not the cause of the increased CLT density. The effect of the paracrine activity of Ang-1 on EC survival and proliferation has been examined by different research groups [28–31], as was the synergy between VEGF and Ang-1 against EC apoptosis [26]. According to our hypothesis, different angiogenic factors use a variety of intercellular signalling cascades, some of which are shared, so their increased concentration (likewise their synergy) is the main effect of increased CLT formation in bilayered substitutes. In addition, increased MMP activity and matrix remodelling contribute to the increase of angiogenic factors and help EC migration and sprout formation.

## 5. Conclusion

Our team developed a vascularized skin substitute approach using the self-assembly method to follow the self-organization behaviour of ECs at different times. We also determined the ideal conditions for constructing complex CLTs in skin substitutes with HDMECs. The expression of different angiogenic factors (VEGF, Ang-1, and MMPs) was increased by seeding keratinocytes without causing inflammation, which resulted in increased endothelial survival, proliferation and migration, and matrix degradation. Our results showed that* in vitro* microvascular network formation in tissue-engineered skin models is predominantly managed by the epidermal component of substitutes.
